# Exploring racial/ethnic differences in substance use: a preliminary theory-based investigation with juvenile justice-involved youth

**DOI:** 10.1186/1471-2431-11-71

**Published:** 2011-08-16

**Authors:** Sarah W Feldstein Ewing, Kamilla L Venner, Hilary K Mead, Angela D Bryan

**Affiliations:** 1 The Mind Research Network, Pete & Nancy Domenici Hall, 1101 Yale NE, Albuquerque, NM, 87106, USA; 2 The University of New Mexico, Department of Psychology, 1 University of New Mexico, MSC03 2220, Albuquerque, NM 87131-1161, USA; 3 Center on Alcoholism, Substance Abuse and Addictions (CASAA), 2650 Yale SE, Albuquerque, NM, 87106, USA

**Keywords:** substance use, adolescent, risk factors, race/ethnicity, juvenile justice

## Abstract

**Background:**

Racial/ethnic differences in representation, substance use, and its correlates may be linked to differential long-term health outcomes for justice-involved youth. Determining the nature of these differences is critical to informing more efficacious health prevention and intervention efforts. In this study, we employed a theory-based approach to evaluate the nature of these potential differences. Specifically, we hypothesized that (1) racial/ethnic minority youth would be comparatively overrepresented in the juvenile justice system, (2) the rates of substance use would be different across racial/ethnic groups, and (3) individual-level risk factors would be better predictors of substance use for Caucasian youth than for youth of other racial/ethnic groups.

**Methods:**

To evaluate these hypotheses, we recruited a large, diverse sample of justice-involved youth in the southwest (N = 651; *M *age = 15.7, *SD *= 1.05, range = 14-18 years); 66% male; 41% Hispanic, 24% African American, 15% Caucasian, 11% American Indian/Alaska Native). All youth were queried about their substance use behavior (alcohol, marijuana, tobacco, illicit hard drug use) and individual-level risk factors (school involvement, employment, self-esteem, level of externalizing behaviors).

**Results:**

As predicted, racial/ethnic minority youth were significantly overrepresented in the juvenile justice system. Additionally, Caucasian youth reported the greatest rates of substance use and substance-related individual-level risk factors. In contrast, African American youth showed the lowest rates for substance use and individual risk factors. Contrary to predictions, a racial/ethnic group by risk factor finding emerged for only one risk factor and one substance use category.

**Conclusions:**

This research highlights the importance of more closely examining racial/ethnic differences in justice populations, as there are likely to be differing health needs, and subsequent treatment approaches, by racial/ethnic group for justice-involved youth. Additionally, this study highlights the need for timely, empirically supported (developmentally and cross-culturally) substance abuse interventions for all justice-involved youth.

## Background

Mainstream American youth exhibit high rates of substance use. Recent studies indicate that an average of 45% of high-school adolescents regularly drink, 19% smoke cigarettes, 20% use marijuana, and 10% use harder illicit drugs [e.g., [1]]. Prior studies have indicated that justice-involved adolescents may demonstrate even greater rates of substance abuse. Substance use disorders (SUDs) have been found to be one of the most prevalent disorders within the juvenile justice system, with almost half of justice-involved youth meeting criteria for at least one SUD [e.g., [2]].

Not only is substance use prevalent among justice-involved youth, it has also been implicated in both justice system involvement as well as longer-term negative consequences. Adolescent substance use has been strongly related to juvenile and adult criminal justice involvement [3,4], various health risk behaviors [e.g., [5,6]], sustained substance use and related consequences [e.g., [7-9]], and poorer life outcomes [e.g., [10-12]].

While concerning on its own, the negative health trajectory initiated by substance use and justice involvement becomes more consequential when the relative representation of the youth in the justice system is considered. To that end, some posit that the racial/ethnic representation of justice-involved youth mirrors the general population [e.g., [13]], while others contend that the racial/ethnic minority youth are significantly overrepresented in justice systems [e.g., [14,15]]. In addition, previous studies have indicated that justice-involved Caucasian adolescents may be more likely to receive substance abuse treatment than minority adolescents [e.g., [16]] and that racial/ethnic minorities may be less likely to receive treatment than to be arrested for substance-related behaviors [17]. This is noteworthy, as mainstream Native American and Hispanic adolescents have been found to match or surpass Caucasian adolescents in terms of alcohol and marijuana use [18-20] and have substantially higher levels of adverse substance-related consequences and co-occurring risk-taking behaviors [20-22]. Moreover, it has been found that justice-involved adolescents are unlikely to disrupt this potential trajectory on their own; as, once released, justice-involved adolescents are unlikely to seek substance use [23] or other mental health interventions [24].

Notably, a complete understanding of the nature of substance use and its contributing factors are not clear across youth of different racial/ethnic minority groups, particularly for those involved in the justice system. While some have found greater levels of substance use among racial/ethnic minority youth, most have found that justice-involved Caucasian youth tend to be more extreme both in terms of substance use as well as in terms of other risk factors [e.g., [14,25-28]]. Yet, the lack of understanding as to what factors may confer risk for racial/ethnic minority youth indicates the need for additional studies to more closely examine this issue with a sample of diverse, justice-involved youth.

There are many factors that may contribute to adolescent substance use. In particular, the literature has indicated the role of variables in three key domains: parent, peer, and individual. While parent and peer variables have been well supported [e.g., [29-31]], in examining potential targets for improving adolescent-focused prevention/intervention efforts it is critical to evaluate individual-level variables. Among other factors, studies have indicated that low school involvement, high employment, low self-esteem, and high externalizing behavior during adolescence are strongly related to adolescent substance use [32-38]. However, the importance of these individual-level risk factors has primarily been examined with mainstream and Caucasian youth.

While there are many likely contributions to and correlates of racial/ethnic differences (e.g., social economic, educational, cultural) in adolescent substance use, the examination of the existence of such differences is an important first step in its own right. Specifically, examining racial/ethnic differences in representation, substance use, as well as their correlates (individual-level risk factors) in the juvenile justice system is critical, as studies suggest that these inequities are linked to differential long-term outcomes for youth of different racial/ethnic backgrounds [39]. Specifically, recent work has highlighted that despite lower levels of substance use, justice-involved racial/ethnic minority youth evidence greater levels of impairment following their justice involvement than their Caucasian peers [39]. Moreover, there is a paucity of knowledge regarding the most efficacious prevention/intervention programming for racial/ethnic minority, high-risk, justice-involved youth (Feldstein Ewing, Wray, Mead: Adapt and evaluate or evaluate and adapt, submitted). Thus, research highlighting potential areas where prevention/intervention efforts could be improved, particularly for racial/ethnic minority youth are of critical importance to improving health outcomes for high-risk youth [e.g., [40]].

### Investigating Racial/Ethnic Differences: Theoretical Approach

While substantial efforts have been devoted towards evaluating racial/ethnic differences, few have been theoretically grounded. In an effort to systematize evaluations of racial/ethnic differences and their potential implications in the field of health, Carter-Pokras and Baquet [41] present a conceptual review that thoroughly explores the existing literature and theory in this area. Through their research, they provide a working definition of health disparities, as well as a theoretical framework to guide the evaluation of their existence. Following their theoretical framework, we took their recommended steps to examine the potential existence of health-related racial/ethnic differences in a large, diverse sample of justice-involved youth. In this study, our goal was to identify targets for better prevention/intervention efforts to improve adolescent health outcomes for justice-involved and minority youth. Specifically, we aimed to evaluate the (1) relative racial/ethnic representation in a system of justice (juvenile probation), (2) relative rates of substance use (alcohol, tobacco, marijuana, and other illicit hard drug use), and (3) the relationships between individual-level risk factors (school involvement, employment, self-esteem, and level of externalizing behaviors) and substance use across racial/ethnic groups. We hypothesized the following: (1) Based upon the research of Braithwaite and colleagues [14], that racial/ethnic minority youth would be comparatively over-represented in the juvenile justice system. (2) Based upon prior studies examining potential racial/ethnic differences in justice-involved youth [e.g., [14,25,26]], we posited that there would be racial/ethnic differences in terms of substance use rates (i.e., greater use among Caucasian youth compared with racial/ethnic minority youth). Finally, (3) as the phenomena of substance use may be less understood for racial/ethnic minority compared with mainstream Caucasian youth, we proposed that a set of individual-level risk factors would be more effective for predicting substance use for Caucasian versus racial/ethnic minority youth.

This study offered a timely opportunity to examine contributing factors to racial/ethnic differences in adolescent substance use with a high-risk sample (justice-involved youth). While there is precedent establishing the existence of racial/ethnic differences among justice-involved youth, the factors that underlie those differences are significantly less well understood. From a clinical standpoint, evaluating the reasons why differences in substance use might exist (through investigating potential risk factors), provides a firm avenue to guide future prevention and intervention efforts for this diverse, and often underserved, group of high-risk youth. Ultimately, research deconstructing differences on contributing factors is necessary in order to interrupt the pattern of negative, long-term health sequelae for justice-involved youth [39].

## Methods

### Participants and Procedures

Data were collected as part of a larger, longitudinal study (PI: ADB). The goal of this study was to obtain a convenience sample of ethnically-diverse adolescents on probation in order to explore issues of risk behavior and its correlates in depth among high-risk youth. Sample size determination based on power calculations was conducted for the larger longitudinal study for which latent growth modeling analyses are eventually planned, and thus our original target for recruitment was 800 young people, of whom we were able to recruit 714. In addition to the recruited youth, an additional 387 youth indicated initial interest in the study, but did not participate for several reasons. Specifically, 18.9% of youth were ineligible due to age, 3.4% entered detention or treatment after providing assent rendering them inaccessible for participation, 28.4% did not provide effective parent contact information (preventing the securing of parental consent), and consistent with other high risk youth studies (Montanaro, Ewell, Bryan, Feldstein Ewing: Challenges associated with longitudinal research with adolescents: A two- sample perspective, submitted), 49.4% lost interest or repeatedly no-showed. This analysis focused on data from the baseline assessment.

The sampling frame was purposefully selected to yield the largest and most diverse justice-involved sample possible from the state of Colorado. Adolescents (aged 14-18) were recruited from two juvenile probation offices in the Denver Metropolitan area (Denver Main and Adams County). As the goal was to recruit a large, ethnically-diverse sample, these two offices were selected because they were the most ethnically-diverse and the most populous in the state. Because Denver Main is much larger than Adams in terms of their caseload, the majority of participants (77%) were from Denver Main. Youth in this study were on probation for a variety of reasons, ranging from legal infractions not warranting detainment to recently finishing a detention sentence and transitioning back to non-detained status. Adolescents received probation for a range of offenses, varying from mild (15.4% of sample; i.e., curfew violation, truancy, possession of drugs, graffiti, and joyriding), to moderate (49.1% of sample; i.e., theft, auto theft, burglary, harassment, and selling drugs), to severe (35.5% of sample; i.e., arson, carrying a weapon, sexual offense, child abuse, assault, and criminal trespassing). Of the 714 probation-involved adolescents recruited, 41% self-identified as Hispanic (*n *= 292), 24% as African American (*n *= 170), 15% as Caucasian (*n *= 108), and 11% as American Indian/Alaska Native (AIAN; *n *= 81), and the remaining 9% of adolescents fell into the categories of Asian American/Native Hawaiian and bi-/multi-racial. To conduct meaningful analyses, we limited the analyses to the four racial/ethnic groups for which we had the best representation (e.g., Hispanic, African American, Caucasian, and AIAN), which subsequently reduced the sample from 714 to 651. All statistics apply to these 651 adolescents. The average age was 15.7 (*sd *= 1.05) and 66% were male. Notably, the racial/ethnic representation of this sample was consonant with other studies recruited from juvenile probation offices in Denver, Colorado [e.g., [42]].

Research assistants were present in the waiting rooms of juvenile probation offices in the participating metropolitan area. They were seated next to a sign advertising the opportunity to participate in a longitudinal research study. Youth waiting for their appointments in the lobby were approached by research staff and asked if they would like more information about the study; if so, this information was given to them. At that time, adolescents were evaluated for inclusion (criteria included age 14-17 years, currently on probation, fluency in English, and cognitively capable of understanding the assent form as assessed by the research assistant). However, consistent with the delineated IRB protocols for vulnerable samples (e.g., youth, justice-involved samples), our IRB-approved procedures precluded any formal data collection prior to the collection of documented parent consent. Probation or juvenile justice staff had no involvement in recruitment. Adolescents signed an informed assent form and gave parent/guardian contact information to the research staff member. According to IRB-approved procedures, the parent/guardian was then called via telephone, was made aware that the phone call was being taped, and was then read the complete informed consent document. The parent/guardian was encouraged to ask any questions regarding the study, and their verbal consent was then given. All tapes were logged and kept to verify consent. All procedures were approved by the Institutional Review Board at the University of Colorado at Boulder. A federal Certificate of Confidentiality was obtained due to the age of the subjects and the sensitivity of the data. Participants were compensated $20 for the one-hour baseline survey.

### Measures

All assessments utilized ACASI (Audio-Computer-Assisted Self-Interview) technology on individual laptop computers. ACASI has proven to be a reliable way to obtain survey data assessing risk behaviors [43]. ACASI technology allows for survey questions to be displayed on a computer screen and for questions to be digitally recorded, allowing respondents to hear spoken questions over headphones while they read the question on the computer screen. One of the main benefits of using ACASI technology is that it has been shown to increase levels of self-reporting, especially with regard to high-risk behaviors [44-46]. Several research groups have found that for high-risk adolescents both literacy and the ability to negotiate even very simple skip patterns are problematic [42,47]. ACASI technology is uniquely accessible to those who have low levels of literacy. ACASI technology further eliminates errors of contingent questioning, as skip patterns are automatically initiated based on a respondent's answers. Finally, although no juvenile justice staff were present in the private office where the participant completed the ACASI survey, a trained research assistant was with the participant at all times to answer questions.

Using ACASI technology, adolescents were asked to complete the following instruments. Following Robbins and Bryan [48], we assessed alcohol use using a subset of questions from the RAPI [49], which were standardized and averaged to form an alcohol quantity and frequency index (α = .73). Specifically, the instructions defined one alcoholic drink as "one beer, one glass of wine, or one serving of hard liquor either by itself or in a mixed drink," and items asked, "In the last 6 months, (1) How often did you consume at least one alcoholic drink?" (answered on a 9-point scale ranging from "never" to "every day"), (2) "How many drinks did you usually have at one time?" (answered on a 10-point scale ranging from "none" to "more than 20 drinks"), and (3) "When you drank alcohol how often did you get drunk?" (answered on a 5-point scale ranging from "never" to "always"). Similarly, like LaChance and colleagues [50], past six-month quantity and frequency of other substance use were assessed with single items (see Table 1 for items and response options). Due to the low base rate of hard drug use, as done in Robbins and Bryan [48], we formed a composite measure where use of each drug other than tobacco, alcohol, and marijuana was coded "1" and answers were summed across the hard drugs assessed (crack/cocaine, LSD, mushrooms, ecstasy, GHB, ketamine, methamphetamine, and heroin), resulting in a measure ranging from 0 (none used) to 8 (all hard drugs used). In addition, age of first use for tobacco and marijuana was queried (response options = ages 0 to 18).

**Table 1:** Relative Rates of Past Six-Month Substance Use and Individual-Level Risk Factors

	Hispanic	Caucasian	African-American	American Indian/Alaska Native	Test for Difference
Alcohol Quantity and Frequency (Abbrev. RAPI)	.20_a _(.77)	.34_a _(.78)	-.07_b _(.81)	.19_ab _(.69)	F(3,430) = 4.19, *p *< .01
Tobacco					
Age at first use	13.00_a _(2.38)	12.15_b _(2.79)	13.21_a _(2.57)	12.68_ab _(2.11)	F(3,418) = 3.21, *p *< .05
Number of cigarettes per day*	3.89_a _(2.33)	5.11_b _(2.31)	4.16_a _(2.33)	4.58_ab _(2.59)	F(3,426) = 6.00, *p *< .001

Marijuana					
Age at first use	11.39_a _(2.44)	11.49_ab _(2.37)	12.23_b _(2.19)	11.69_ab _(2.27)	F(3,548) = 3.93, *p *< .01
Frequency of marijuana use**	5.79 (2.66)	6.23 (2.71)	5.69 (2.77)	5.54 (2.93)	F(3,388) = .85, *p *= .47

Hard Drug Composite					
Number of hard drugs used	.61_a _(1.26)	1.05_b _(1.49)	.20_c _(.58)	_._69_ab _(1.38)	F(3,633) = 11.31, *p *< .001

In School (% yes)	79.3% _a_	72.2% _b_	92.4% _c_	76.5% _b_	χ²(3, *n *= 649) = 21.38, *p *< .001

Working (% yes)	20.9%	20.4%	18.8%	22.0%	χ²(3, *n *= 651) = .46, *p *= .93

Self esteem	3.32 _ab _(.50)	3.24 _b _(.56)	3.43_a _(.45)	3.32 _ab _(.50)	F(3,647) = 3.62, p = .01
Reliability by group (α)	.72	.83	.71	.79	

Externalizing behavior	1.59 _a _(.29)	1.72 _b _(.29)	1.60_a _(.31)	1.75 _b _(.31)	F(3,647) = 9.85, p < .001
Reliability by group (α)	.88	.87	.88	.88	

Note: Means not sharing the same subscript are significantly different at p < .05 controlling for experimentwise error rate. All standard deviations are represented in parentheses ( ) underneath the respective mean value.

*1-10 scale where 1 = none, 2 = 1 cigarette, 3 = 2-3 cigarettes, 4 = 4-6 cigarettes, 5 = 7-9 cigarettes, 6 = 10-12 cigarettes, 7 = 13-15 cigarettes, 8 = 16-18 cigarettes, 9-19-20 cigarettes, 10 = more than 20 cigarettes

**1-8 scale where 1 = occasionally, 2 = once a month, 3 = 2-3 times a month, 4 = 4-5 times a month, 5 = once a week, 6 = 2-3 times a week, 7 = 4-5 times a week, 8 = every day

In terms of individual-level risk factors (see Table 1), to assess school involvement and current employment, adolescents were asked whether they were currently enrolled in school and currently working, with a yes/no response option for each. Self-esteem was measured with an 8-item version of Rosenberg's [51] Self-esteem Scale [e.g., [52]]. Externalizing behavior was measured with the Youth Self-Report [CBCL YSR; 53], which has well established reliability and validity. These measures evidenced acceptable reliability in this study (Self-esteem α = .75; Externalizing behavior α = .88) and were normally distributed based on skew and kurtosis near zero for both scales.

### Analysis Plan

To evaluate *Hypothesis 1 *(i.e., that racial/ethnic minority youth would be comparatively over-represented in the juvenile justice system), we compared the racial/ethnic demographics of the relevant metropolitan area to the representation in this probation sample. To evaluate *Hypothesis 2 *(that justice-involved Caucasian youth would have greater rates of substance use than ethnic minority youth), we explored differences in this sample's substance use across racial/ethnic groups using ANOVA for continuous measures and χ^2 ^tests for categorical measures. To evaluate *Hypothesis 3 *(that individual-level risk factors would be more effective for predicting substance use for Caucasian versus racial/ethnic minority youth), we examined the differences in individual-level risk factor by racial/ethnic group using ANOVA for continuous measures and χ^2 ^tests for categorical measures. Then, we tested whether the relationship of risk factors to substance use was consistent across racial/ethnic groups. Varying degrees of freedom across analyses reflect adolescents who did not report use of a particular substance, sporadic missing data on particular questions due to participants opting out of answering an item, or to a question being added after study initiation. We analyzed all available data for each particular analysis. All reported tests utilize two-tailed alpha and all posthoc tests following a significant overall effect are corrected for alpha inflation using the Bonferonni method since we did not have strong hypotheses regarding the direction of these effects.

## Results

### Relative Representation in a System of Justice

According to the U. S. Census [54], the participating metropolitan area has the following demographic distribution: 20% Hispanic, 4% African American, 71% Caucasian, and 1% AIAN. In contrast, the self-identified racial/ethnic distribution of this juvenile probation sample was: 41% Hispanic, 24% African American, 15% Caucasian, and 11% AIAN.

### Relative Rates of Current (Past Six-Month) Substance Use

Similar to previous studies [e.g., [14,55,56]], the most common substances used by this sample were alcohol, marijuana, and tobacco (approximately 70% each). For alcohol, adolescents reported drinking on average approximately once per month; 31% did not drink at all and 21% drank at least weekly. Those who drank consumed an average of 4-6 drinks per occasion. When asked how often they got drunk, 42% of those who drank said they "almost always" or "always" got drunk. Of the 430 cigarette smokers, the average number of cigarettes smoked per day was 4-6. Marijuana users (n = 391) reported using marijuana an average of 2-3 times per week, and 48% of marijuana users reported smoking every day. Only 29% of the sample reported hard drug use; the most frequently used hard drugs included ecstasy (18%), mushrooms (15%), and crack-cocaine (12%). Ethnic/racial differences emerged on all substance use categories except frequency of marijuana use (see Table 1).

In terms of racial/ethnic differences in substance use, African American adolescents had the lowest alcohol use, which was significantly lower than Caucasian youth. For frequency of alcohol use and frequency of getting drunk, AIAN adolescents did not differ from other groups; they fell between African American and Caucasian youth. Of the 433 cigarette smokers, the average number of cigarettes smoked per day was 4-6. Caucasian youth were younger at age of first cigarette than Hispanic and African American youth. Again, AIAN youth's age at first use fell between Caucasians versus Hispanic and African American youth. Caucasian adolescents smoked significantly more cigarettes than Hispanic youth. Marijuana users (n = 392) reported using marijuana an average of 2-3 times per week and no differences in frequency emerged. However, Caucasian youth were significantly younger at first marijuana use than African American youth. In terms of hard drug use, African American youth used far fewer, while Caucasian youth used significantly more hard drugs than any other ethnic group. Hispanic and AIAN adolescents did not differ in the number of hard drugs they had used.

### Relative Rates of Individual-Level Risk Factors

There were no significant differences by racial/ethnic group in terms of percent employed. There were significant group differences in school enrollment, self-esteem, and externalizing behavior (see Table 1). In terms of racial/ethnic differences, African American youth were significantly more likely than all other groups to be enrolled in school, and Hispanic youth were more likely to be in school than Caucasian or AIAN youth, who did not differ. Further, African Americans and Hispanic youth had the highest self-esteem and the lowest externalizing scores; Caucasian and AIAN adolescents had lower self-esteem and higher externalizing scores, but did not differ.

### Relationships between Individual-Level Risk Factors and Current Substance Use by Racial/Ethnic Group

Table 2 contains the correlations between risk factors and substance use. School enrollment was associated with significantly less alcohol and hard drug use. Higher self-esteem was associated with lower frequency of hard drug use. Higher externalizing behavior was associated with greater quantity and frequency of alcohol, marijuana, cigarettes, and hard drug use.

**Table 2:** Relationships between Individual-Level Risk Factors and Past Six-Month Substance Use

	Alcohol quantity and frequency	Number of cigarettes smoked per day	Marijuana frequency	Number of hard drugs used
Enrolled in school	-.15**	-.18***	-.05	-.14***
Self-esteem	-.08	-.07	-.04	-.14***
Externalizing behavior	.32***	.22***	.14**	.27***

*p < .05, **p < .01, ***p < .001

Our final analysis examined interactions between individual-level risk factors and racial/ethnic group. As described in the Analysis Plan, we used analytic techniques based upon the distribution of each outcome variable. Because of the number of tests, critical alpha was adjusted to .01. We did not use Bonferonni due to the stringency of that correction [see 57], as these were exploratory analyses meant to generate questions for further research. One significant interaction emerged. Because our hard drug use variable was right-skewed, we utilized generalized estimating equations in SAS (PROC GENMOD procedure) allowing for the specification of a Poisson-distributed outcome. To test the interaction, we followed outlined procedures [58] for testing categorical by continuous interactions. First, externalizing behavior was centered so that the mean was zero. Then, dummy codes were created for each non-Caucasian race/ethnic group utilizing Caucasians as the reference. The centered externalizing behavior variable was multiplied by each of the three dummy codes so a total of three terms were added to the equation. In this analysis, the externalizing behavior × African American interaction effect was significant (est.= -1.60, 95% confidence limits [-2.8711 to -0.3278], *p *= .01). Specifically, the effect of externalizing behavior on hard drug use differed for African American versus Caucasian youth. The Spearman correlations between externalizing behavior and hard drug use for the four groups were: Hispanic - ρ = 0.31, *p *< .001; Caucasian - ρ = 0.41, *p *< .001; African American - ρ = 0.15, *ns*; and AIAN - ρ = 0.33, *p *< .01. The relationship between externalizing behavior and hard drug use was strong and significant among Caucasian adolescents, and this did not differ for Hispanic and AIAN adolescents. However, there was no association between externalizing behavior and hard drug use for African American youth.

## Discussion

Following the theory-based approach of Carter-Pokras and Baquet [38] guiding the evaluation and deconstruction of racial/ethnic differences in the field of health, this preliminary study sought to evaluate the existence of racial/ethnic differences among justice-involved youth by examining the recommended three contributing factors: (1) relative representation in a system of justice (juvenile probation), (2) relative rates of substance use (alcohol, tobacco, marijuana, and other illicit hard drug use), and (3) the relationships between a set of individual-level risk factors (school involvement, employment, self-esteem, and level of externalizing behaviors) and substance use across racial/ethnic groups.

More specifically, in this study, we found support for Hypothesis 1, the over-representation of racial/ethnic minority youth in the juvenile justice system. In contrast to recent surveys [13], this sample contained a disproportionate representation of racial/ethnic minority youth [54]. Specifically, as compared to the participating metropolitan area, there were twice as many Hispanic youth, six times as many African American youth, ten times as many AIAN youth, and only one-fifth as many Caucasian youth as would be expected [54].

For Hypothesis 2 we posited that justice-involved Caucasian youth would evidence greater substance use rates than racial/ethnic minority youth. This hypothesis was supported, as Caucasian adolescents evidenced the greatest rates across almost all substance use categories. In contrast to the broader adolescent literature which found the greatest levels of use among AIAN and Hispanic youth [18-20], these findings are consistent with other literature sampling justice involved youth [e.g., [14]]. In terms of other racial/ethnic differences, African American adolescents demonstrated the lowest rates of use, and AIAN and Hispanic youth generally fell in between those two groups. Together, these data support that despite their respective underrepresentation in the justice system, justice-involved Caucasian adolescents may have much greater rates of substance use than adolescents of other racial/ethnic groups [e.g., [25-28]].

In terms of Hypothesis 3, as the level of substance use in this sample was significant enough to potentially influence these youths' later health outcomes [1], we examined individual-level risk factors that could predict substance use. Specifically, we evaluated whether these risk factors were more effective in predicting substance use for Caucasian versus racial/ethnic minority youth. Consistent with prior studies that have found that Caucasian youth may be more severe in terms of psychopathology [e.g., [59]], we found that comparatively, Caucasian youth evidenced the greatest risk. In addition, African American youth evidenced the lowest risk, and AIAN and Hispanic youth fell between Caucasian and African-American youth. Specifically, Caucasian youth had higher externalizing behavior scores than African American or Hispanic youth, as well as significantly lower levels of self-esteem than African American youth. Contrary to predictions, a racial/ethnic group by risk factor finding emerged for only one risk factor and one substance use category; externalizing behavior was a significant correlate of hard drug use for Caucasian youth, but showed no relationship to hard drug use for African American youth. These data indicate that, with respect to Hypothesis 3, that the frequently employed individual-level risk factors were less useful in flagging potential points of concern for the high risk youth of this sample, particularly for African American youth.

While not predicted, the most profound differences emerged between African American and Caucasian youth. One potential explanation for this difference is that more protective factors may exist in our society to prevent Caucasian youth's justice involvement. For example, while not explicitly examined in this study, studies have suggested that high-risk and substance-abusing Caucasian youth may be "referred" to hospitals, medical care, and treatment, whereas African American and other racial/ethnic minority youth are alternatively "referred" to justice settings [60]. Additionally, as found with other studies, African American youth in this sample had the lowest level of overall substance use [e.g., [61]], but were disproportionately represented in this sample. These findings indicate the need for better prevention of justice involvement, as well as better indicators of what might be points of risk (e.g., measurements of potential individual-level risk factors) for African American youth.

Together, these data indicate support for two of the three points of evaluation recommended by Carter-Pokras and Baquet [38]. To that end, these findings support the existence of racial/ethnic differences in the relative representation and patterns of substance use of justice-involved adolescents. Additionally, these data suggest that the current empirically-supported avenues to identify youth who might be at-risk for abusing substances (e.g., measurements of individual level risk factors) may be less effective for indicating the need for intervention among justice-involved youth, and particularly among racial/ethnic minority youth within this context. This is highly relevant, as racial/ethnic minority youth have been found to evidence greater functional impairment in the years following justice involvement [39]. Together, these data indicate the importance of continuing to identify and evaluate risk factors for justice-involved and racial/ethnic minority youth, as well as the need to adapt and/or create measurement approaches to effectively evaluate these constructs among justice-involved minority youth. Finally, consonant with the theoretical approach of Carter-Pokras and Baquet [38], an important next step to follow this preliminary study is to evaluate whether or not the observed racial/ethnic differences are avoidable and unfair; this is key to determining the existence of inequity.

In terms of clinical implications, to effectively address the diverse health needs of youth entering the justice system, these findings highlight the need for timely, developmentally-, and culturally-appropriate substance abuse interventions to all youth entering the justice system [e.g., [16]]. One brief intervention which has shown promise in high-risk youth and cross-cultural applications is motivational interviewing [e.g., [62]]. Similarly, as the picture of health needs that these youth may face may be more complex than what might be addressable within the justice system, these data also suggest that comprehensive, multi-level services, such as the Wrap-around work pioneered by Karl Dennis [e.g., [63]], might be uniquely able to address the differing, but equally important needs of these high-risk youth. Finally, in terms of potential avenues for prevention, these data highlight the importance of determining where differences (and potential inequities) may begin in order to guide more effective prevention programming for these high-need youth. Specifically, if there are indeed differences in the relative rates of referral [60], it will be important to determine the level at which the differential referral may be taking place (e.g., community mental health providers, social service agencies, schools, local law enforcement agencies), to guide public policy and educational programming changes. Furthermore, while many research groups (including our own) are actively evaluating health risk trajectories and outcomes among high-risk youth (Bryan, Schmiege, Magnan: Marijuana use and risky sexual behavior among high risk adolescents: Trajectories, risk factors, and event-level relationships, submitted; Feldstein Ewing, Schmiege, Bryan: Continued detention involvement and adolescent marijuana use trajectories, submitted), greater attention needs to be paid to the front end of this continuum. In particular, research needs to continue to explicitly evaluate contributing factors and patterns of substance use with high-risk racial/ethnic minority youth to identify places to most effectively target prevention and intervention approaches. This important work will help highlight how to tailor and implement prevention approaches with high-risk youth in order to improve youth's long-term health outcomes [64].

## Conclusions

This study employed the theory-based approach of Carter-Pokras and Baquet [38] to evaluate the existence of racial/ethnic differences in adolescent health among justice-involved youth by examining three contributing factors. Confirming the first hypothesis, this study revealed a disproportionate representation of racial/ethnic minority youth within this justice sample [54]. Specifically, as compared to the participating metropolitan area, there were twice as many Hispanic youth, six times as many African American youth, ten times as many AIAN youth, and only one-fifth as many Caucasian youth as would be expected [54]. Second, in examining the relative rates of substance use (alcohol, tobacco, marijuana, and other illicit hard drug use), this study found significant differences between racial/ethnic groups, with the greatest rates of use among Caucasian youth, the lowest rates of use among African American adolescents, and with AIAN and Hispanic youth in the middle. Third, this study examined the relationship between individual-level risk factors (school involvement, employment, self-esteem, and level of externalizing behaviors) and substance use across racial/ethnic groups. Similar to the rates of substance use, Caucasian youth evidenced the greatest risk and African American youth demonstrated the lowest risk, with AIAN and Hispanic youth in between. Contrary to predictions, a racial/ethnic group by risk factor finding emerged for only one risk factor and one substance use category; externalizing behavior was the best predictor of hard drug use for Caucasian youth. In sum, this research supports the utility of employing a theory-based framework to examine racial/ethnic differences and their potential implications, and highlights the importance of future research to more closely scrutinize potential individual-level risk factors among racial/ethnic minority youth and to determine measurement approaches to effectively evaluate these potential risk factors for minority youth. Additionally, this study highlights the continued importance of examining racial/ethnic differences in justice populations, as there are likely to be differing health needs and effective avenues for intervention by racial/ethnic group for justice-involved youth.

### Limitations and Future Directions

The strengths of this study include the theory-based approach, large sample size, the significant representation of multiple racial/ethnic groups to support the cross-groups analysis, the comprehensive evaluation of substance use and identified risk factors, and the focus on the health issues of justice-involved youth. However, the findings of this study should be interpreted in light of the following limitations. To address the study questions, this study employed a convenience sample, resulting in the attendant limitations in terms of generalizability; future replication with a larger, nationally representative sample of adolescents involved with the juvenile justice system will be critical to following up with and further exploring the questions posed in our research. This was a cross-sectional study; future work should examine substance use progressions in a longitudinal design in order to strengthen our ability to draw causal conclusions regarding risk factors for progression of substance use. Additionally, consistent with the delineated IRB protocols for vulnerable samples (e.g., youth, justice-involved samples), our IRB-approved procedures precluded data collection on youth prior to the collection of documented youth assent and parent consent. Therefore, we were unable compare exact characteristics of those who participated versus those who did not. Moreover, the selection of risk factors was limited to individual-level risk factors believed to influence potential racial/ethnic differences; future work should examine the comparative influence of salient parent and peer factors, as well as critical environmental and genetic factors [e.g., [65]]. Finally, although the sample was sizeable, the numbers of specific racial/ethnic groups, particularly AIAN and African American individuals, was relatively small, perhaps limiting generalizability. Race and ethnicity were assessed in a mutually exclusive fashion, and this procedure differs from those currently employed in many large-scale surveys (e.g., the Census). Additionally, limited data were collected with respect to cultural factors that relate to race and ethnicity. Thus, important information relating to culture and substance use behaviors were not considered in the present analyses. Future studies are needed to address these limitations.

## Competing interests

None of authors have financial or non-financial competing interests that would influence the interpretation of the data or presentation of the information provided in this report.

## Authors' contributions

SWFE conceptualized, designed, and drafted the manuscript. KLV participated in the conceptualization and the design of the project, and assisted with editing the manuscript. HKM assisted in the editing of the manuscript. ADB designed the longitudinal study from which this data were drawn, assisted with the conceptualization and design of the manuscript, carried out analyses and assisted with the editing of the manuscript. All authors read and approved the final manuscript.

## Author's information

SWFE is an Assistant Professor of Translational Neuroscience at the Mind Research Network (Albuquerque, NM, USA). AB is a Professor of Psychology at the University of New Mexico (Albuquerque, NM, USA). KLV is an Assistant Professor of Psychology at the University of New Mexico/Center for Alcohol and Substance Abuse Addictions (CASAA; Albuquerque, NM, USA). HKM is a postdoctoral fellow at the Mind Research Network (Albuquerque, NM, USA).

## Pre-publication history

The pre-publication history for this paper can be accessed here:

http://www.biomedcentral.com/1471-2431/11/71/prepub
